# Supplementary material for: Biomarkers associated with cell-in-cell structure in kidney renal clear cell carcinoma based on transcriptome sequencing
Source: PeerJ. 2025 Apr 16;13:e19246. doi: 10.7717/peerj.19246 (PMC12009028; doi:10.7717/peerj.19246)
Supplement: Supplemental Information 1 [file peerj-13-19246-s001.docx]

Supplementary Table 1. Primer sequences used in this study.

| Gene | Accession No. | Primers (5’-3’) | |
| --- | --- | --- | --- |
|  |  | Forward | Reverse |
| *TGFB1* | NM_000660 | CCGACTACTACGCCAAGGAGGT | TCAACCACTGCCGCACAACTC |
| *CDKN2A* | NM_058195 | TACTGAGGAGCCAGCGTCTAGG | ACCACCAGCGTGTCCAGGAA |
| *CDC20* | NM_001255 | TGTCTGAGTGCCGTGGATGC | GTCTTCAGCGGATGCCTTGGT |
| *CTSS* | NM_004079 | TGTGCTCTTGGTGTGCTCCTCT | GGCTGGGAACTCTCAGGGAACT |
| *VIM* | NM_003380 | CTGAATGACCGCTTCGCCAACT | TCCCGCATCTCCTCCTCGTAGA |
| *GAPDH* | NM_002046 | GTCTCCTCTGACTTCAACAGCG | ACCACCCTGTTGCTGTAGCCAA |
